# Inferred father-to-son transmission of herpes simplex virus results in near-perfect preservation of viral genome identity and *in vivo* phenotypes

**DOI:** 10.1038/s41598-017-13936-6

**Published:** 2017-10-20

**Authors:** Utsav Pandey, Daniel W. Renner, Richard L. Thompson, Moriah L. Szpara, Nancy M. Sawtell

**Affiliations:** 1 Department of Biochemistry and Molecular Biology, Center for Infectious Disease Dynamics, and the Huck Institutes of the Life Sciences, Pennsylvania State University, University Park, Pennsylvania 16802 USA; 2 Department of Molecular Genetics, Biochemistry and Microbiology, University of Cincinnati, Cincinnati, Ohio 45229 USA; 3 Division of Infectious Diseases, Cincinnati Children’s Hospital Medical Center, Cincinnati, Ohio 45229 USA

## Abstract

High throughout sequencing has provided an unprecedented view of the circulating diversity of all classes of human herpesviruses. For herpes simplex virus 1 (HSV-1), we and others have previously published data demonstrating sequence diversity between hosts. However the extent of variation during transmission events, or in one host over years of chronic infection, remain unknown. Here we present an initial example of full characterization of viruses isolated from a father to son transmission event. The likely occasion of transmission occurred 17 years before the strains were isolated, enabling a first view of the degree of virus conservation after decades of recurrences, including transmission and adaptation to a new host. We have characterized the pathogenicity of these strains in a mouse ocular model of infection, and sequenced the full viral genomes. Surprisingly, we find that these two viruses have preserved their phenotype and genotype nearly perfectly during inferred transmission from father to son, and during nearly two decades of episodes of recurrent disease in each human host. Given the close genetic relationship of these two hosts, it remains to be seen whether or not this conservation of sequence will occur during non-familial transmission events.

## Introduction

Herpes simplex viruses (HSV) are widespread human pathogens. HSV-1 and HSV-2 together have ~90% incidence worldwide^1^. HSV infections are a major public health concern, causing mucocutaneous and systemic diseases^2^. In the USA, HSV-1 is the leading cause of sporadic necrotizing encephalitis and infectious blindness^3,4^. HSV infection remains a significant cause of morbidity and mortality in neonates, with an incidence of approximately 1 per 3,200 deliveries in the United States^5,6^. HSV-2, which has 70% genomic similarity to HSV-1, is associated with higher HIV/AIDS acquisition in developing countries^7–9^. Traditionally, epidemiologically related strains of HSV-1 or HSV-2 have been identified by comparing restriction fragment length polymorphism (RFLP) patterns or using targeted Sanger sequencing of selected genes or loci^10–13^. Although RFLPs were crucial to establish our overall understanding of HSV transmission, the limited number of nucleotides assayed by changes in RFLP pattern obscured variation at the level of single nucleotide variations (SNVs) and small insertions or deletions (in/dels). Advancements in high-throughput sequencing (HTS) have now made it possible to study the full extent of genetic variation in the viral population being transmitted between different hosts.

A transmission event has the potential to create a bottleneck that reduces genetic variation, if only a limited number of founder viruses are transmitted to the next host^14–18^. Viral genetic diversity may be created *de novo* through replication, recombination, and/or selection in the new host, leading to a viral population that is genetically distinct from the founder population. Most transmission studies have focused on RNA viruses, which have a larger amount of standing variation in the population^19,20^. Less is known about bottlenecks or expansion of variation during transmission of large DNA viruses. The transmission bottleneck has been shown to be more restrictive for RNA viruses than for DNA viruses^17,18^. In the case of human immunodeficiency virus (HIV) and hepatitis C virus (HCV), it is estimated that as few as 1–5 virions may be transmitted between hosts to establish infection^14–16^. In contrast, the founder population for human cytomegalovirus (HCMV), a β-herpesvirus, was estimated to be in the tens to hundreds of virions for maternal-to-fetal transmission^17^. While the conservation of genetic diversity is thought to be more likely during transmission of DNA viruses, data on viral genetic diversity during human-to-human transmission of HSV-1 has thus far been lacking.

Here we present the genetic and phenotypic characterization of HSV-1 isolates obtained from a father and his son. These isolates were obtained from oral lesions of each host, nearly two decades after the inferred transmission and primary infection of the son. We investigated the pathogenicity of these isolates in a mouse ocular model of infection, and found a similar level of replication at the site of inoculation, and a similar level of neuroinvasiveness. The viral isolates were also alike in their ability to establish latency and their rate of reactivation in both *in vivo* and *in vitro* models. We subjected the viral population of each cultured viral isolate to HTS, and found that the genomes were nearly identical at the consensus level. A small number of minority variants were found in each isolate, most of which result from length variations in homopolymeric tracts or short sequence repeats (SSRs). In the light of recent studies showing extensive intra-host variation and rapid evolution during transmission of HCMV^17,21^, the extent of conservation we observed between the isolates was exceptional. This study presents the first example of comprehensive characterization of founder and transmitted HSV-1 isolates using HTS and *in vivo* models of pathogenesis.

## Results

### Familial transmission and viral culture characteristics

**Figure 1:**
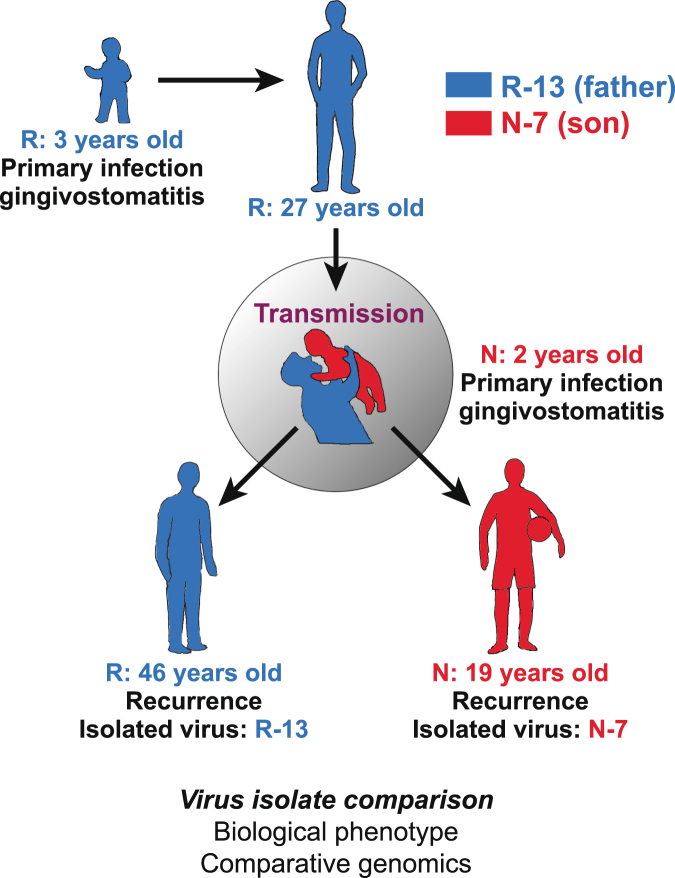
Timing of father to son transmission of HSV and viral isolate acquisition. HSV-1 isolate R-13 was acquired during an orolabial recurrence from a father with lifelong chronic infection, characterized by oral lesion recurrences 6–8 times per year. The father acquired his primary infection at age 3, more than four decades prior. At age 27, the father is inferred to have transmitted HSV-1 to his 2-year old son, who experienced gingivostomatitis with high fever. After resolution of the primary infection, the son likewise experienced recurrent orolabial lesions at a frequency of 6–8 episodes/year. Isolate N-7 was acquired during an orolabial recurrence from the son 17 years after his primary infection. The isolates were obtained on separate occasions, two years apart.

The likely timing of horizontal transmission from father to son, and the collection of the virus isolates from each, is shown schematically in Fig. 1. Both the father and the son experience recurrences 6–8 times per year. The father harbored the virus for 24 years before the likely transmission to the son. The timing of transmission is inferred from the son’s primary episode of gingivostomatitis. The mother was seronegative, and the child had no contact with other caregivers or siblings before this time. Virus was isolated from a recurrence in the father 43 years after his primary infection (isolate R-13), and from a recurrence in the son 17 years after the primary infection. The isolates from father and son were collected on independent occasions, two years apart. No differences in plaque size or morphology were noted between the isolates upon culturing. Both virus isolates were found to replicate with equivalent efficiency. RFLPs were examined in a series of Southern blots on DNA cleaved with several different restriction enzymes, utilizing diverse simple probes (~1 kb) or complex cosmid probes of 27 to 45 kb in length that spanned the entire viral genome (see Supplementary Fig. 1 for a representative example). At this level of resolution, no genetic differences were evident between the two isolates.

### Acute replication kinetics and mortality of HSV-1 isolates R-13 and N-7

**Figure 2:**
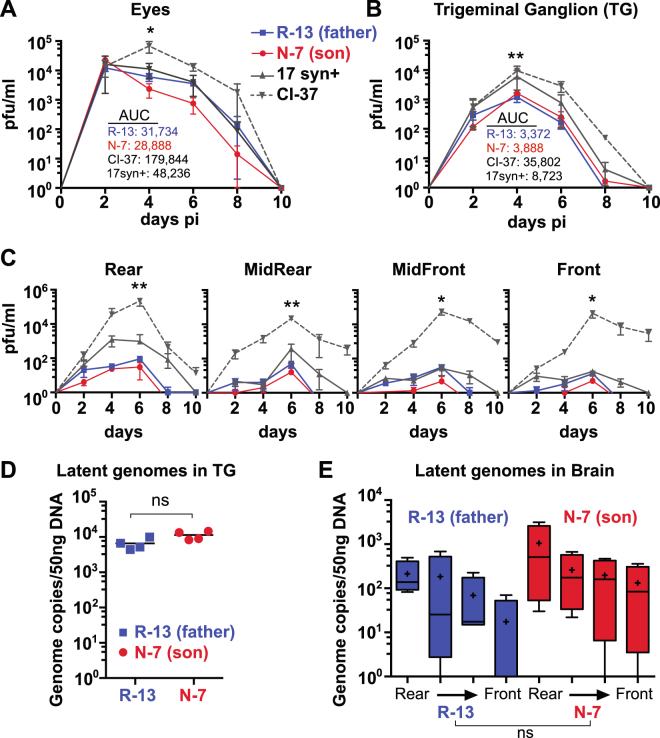
Quantification of replication and latency phenotypes of R-13 and N-7 during infection *in vivo*. Swiss Webster mice (male) were infected on scarified corneas with 2 × 10^5^ pfu of the clinical isolate R-13 or N-7. At the indicated times post-infection, tissues collected from each of three mice per group were assayed for infectious virus using a standard plaque assay (see Methods for details). Replication in eyes **(A)** and TG **(B)** revealed that infectious virus generated in these tissues during the acute stage of infection was not significantly different between R-13, N-7, or 17syn+ , whereas the levels for CI-37 were significantly higher (Student’s t test, *p ≤ 0.05, **p ≤ 0.01, for peak titer on day 4; AUC = area under the curve) **(C)** The replication kinetics and regional distribution of isolates in the brain was determined by cutting each brain into 4 coronal sections. The levels of infectious virus were not significantly different between R-13, N-7, or 17syn+, whereas the levels for CI-37 were significantly higher (Student’s t test, *p ≤ 0.05, **p ≤ 0.01, for peak titer on day 6). This low level of infectious virus for R-13 and N-7 in the brain (≤100 PFU) is consistent with the 100% survival observed for both isolates under these infection conditions, whereas CI-37 induced complete mortality (Supplementary Fig. 2). **(D**,**E)** Quantification of latent viral genomes. At 40 days post infection, the TG and brains from R-13 (n = 4) and N-7 (n = 4) latently infected mice were assayed for viral genome copies using real time qPCR. Viral genome copy numbers per 50 ng mouse DNA detected in R-13 and N-7 were not significantly different (Student’s t test) **(D)**. Brains were cut into four coronal sections prior to isolating DNA and the number of viral genomes copies was determined in each section. Viral genome copies in the brains were not significantly different (ANOVA; on box and whisker plot, +indicates mean, bar indicates median) **(E)**.

The *in vivo* pathogenesis properties of the isolates were examined in the mouse ocular model of infection. Groups of outbred male Swiss Webster mice were infected via the cornea with 2 × 10^5^ PFU of father and son isolates R-13 and N-7, the unrelated clinical isolate CI-37, or the HSV-1 reference strain 17syn+ (see Methods for details). At two day intervals over a 10 day period, tissues were harvested from three mice in each group and the virus content quantified by standard plaque titration assays. Replication kinetic curves are shown in Fig. 2A–C. There was no significant difference in peripheral replication on the eye between the father-son viral isolates or HSV-1 17syn+, however the viral titer for CI-37 was significantly higher (Fig. 2A) (p ≤ 0.05; see Fig. 2 for summed value of each area under the curve (AUC)). Likewise, virus replication in trigeminal ganglia (TG) was not significantly different between R-13, N-7 and 17syn + , but was significantly higher for isolate CI-37 (Fig. 2B) (p ≤ 0.01; see Fig. 2 for AUC values). In the ocular model of infection, the central nervous system (CNS) is infected by retrograde transport of the virus from infected neurons in the TG to the trigeminal nucleus in the hindbrain. From there, replicating virus is transported toward the front of the brain through time. To capture some of this process, brain tissue was divided into four roughly equal parts for assay (rear, mid rear, mid front, front) as detailed previously^22^. No significant differences were observed between the father and son viral isolates or HSV-1 17syn + in any of the brain regions, whereas CI-37 had a significantly higher titer in all four brain regions (Fig. 2C) (p ≤ 0.01 for rear and mid rear regions, p ≤ 0.05 for mid front and front regions; see Fig. 2 for AUC values). The amount of virus detected in the brain for R-13 and N-7 was low, reaching just 100 PFU in the rear portion of the brain and less in the front portions. In contrast, the viral isolate CI-37 reached much higher titers of 10^4^ to 10^5^ PFU in each brain region, while 17syn + had an intermediate phenotype. These data were consistent with the 100% survival observed with both R-13 (n = 35) and N-7 (n = 34), the complete mortality of CI-37 (n = 5), and the 81% survival of 17syn + (n = 16) (Supplementary Fig. 2). No signs of encephalitis in either R-13 or N-7 infected groups were observed, although moderate to severe blepharitis did occur.

Since survival rates were high for the father-son isolates R-13 and N-7, additional mice were maintained for 40 days post-infection (p.i.) and TG and brain tissues were analyzed for the presence of the latent viral genomes by quantitative PCR (see Methods for details). There was no significant difference in the number of viral genomes detected in mouse TG that were latently infected with either R-13 or N-7 (Fig. 2D). Likewise, no significant difference in the amount of latent viral DNA present in the four regions of the brain was detected (Fig. 2E). These results were consistent with the prior observation that the isolates replicated equivalently in these tissues.

**Figure 3:**
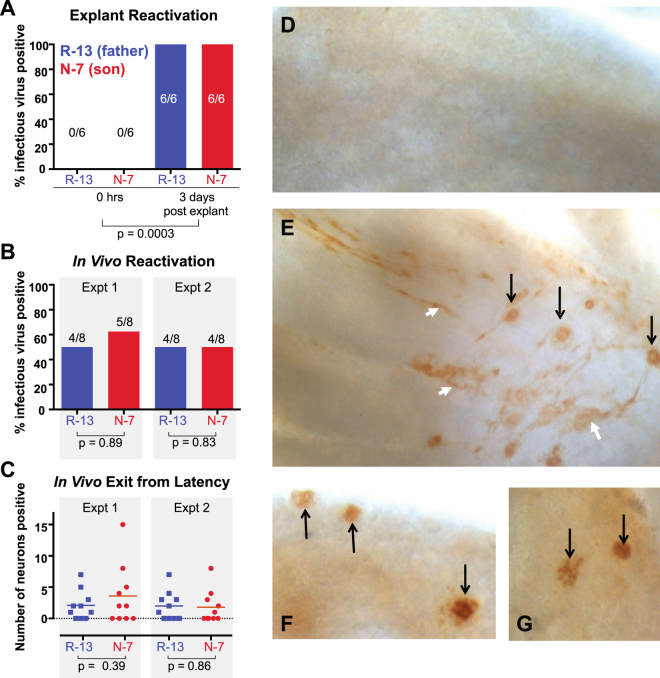
Explant and *In vivo* reactivation in Swiss Webster mice latently infected with R-13 and N-7. R-13 and N-7 were compared for reactivation from latency (>40 days pi) using *in vitro* and *in vivo* reactivation assays. **(A)** In a standard TG explant assay, no difference between R-13 and N-7 reactivation frequency was observed (p = 0.99, Student’s t test) although the difference between virus recovered at time 0 and 3 days post explant was significant in both groups (p = 0.0003, ANOVA). **(B)** The *in vivo* reactivation frequency (percentage of mice with infectious virus detected in TG) was also not different between R-13 and N-7 at 22 hrs. post hyperthermic stress (Student’s t test, expt. 1 p = 0.89, expt. 2 p = 0.83) and **(C)** the number of neurons exiting latency was also not different (expt. 1 p = 0.39, expt. 2 p = 0.86). Latently infected TG were subjected to whole ganglion immunohistochemistry to detect viral protein at 0 hrs. **(D)**, and 3 days **(E)** post explant. Viral protein-expressing neurons (black arrows) and tracts (white arrowheads) mark the range of viral spread. Viral protein-expressing neurons (black arrows) are detectable in TG from N-7 **(F)** and R-13 **(G)**, which reactivated from latently infected TG, at 22 hrs. after hyperthermic stress *in vivo*.

The remaining latently-infected mice were employed to test the ability of the isolates to reactivate from latency. Reactivation was tested after explantation of TG into culture *in vitro* ^23^ or after hyperthermic stress (HS) *in vivo* ^24–26^. No infectious virus was detected in any of six TG tested at 0 hours post-explant in either group, demonstrating that persistent infection or frequent spontaneous reactivation events were absent for both R-13 and N-7^27^ (Fig. 3A and D). After three days in culture all TG samples were positive for virus (Fig. 3A). We conclude from this that latent virus capable of reactivating was present in all TG tested. Whole ganglion immunohistochemical (IHC)^28,29^ detection of viral proteins revealed regions of virus spread within the TG at this time (Fig. 3E). Two separate experiments showed similar efficiency of viral reactivation *in vivo* in TG 22 hrs. post HS, with 50% of the TG examined in both groups containing detectable infectious virus (Fig. 3B). A similar number of TG neurons were found to be expressing viral proteins at 22 hrs. post HS, as detected by whole ganglion IHC (Fig. 3C and F,G). Combined with the finding above that similar numbers of latent viral genomes were present in these TG, we concluded that the relative efficiency of reactivation from latency *in vivo* was also similar between the father-son isolates R-13 and N-7.

### Nearly identical genomes of father and son HSV-1 isolates

To further test the observation of overall genomic and phenotypic similarity of the HSV-1 isolates from father (R-13) and son (N-7), we used HTS and *de novo* assembly approaches to construct a full-length genome sequence for each viral isolate. Viral nucleocapsid DNA was used as the starting point for Illumina paired-end sequencing, with *de novo* assembly and genome annotation carried out using a recently described open-source viral genome assembly (VirGA) workflow^30^ (Table 1) (see Methods for details). At the level of consensus genomes, HSV-1 isolates R-13 and N-7 are 98.9% identical in their DNA sequence (Table 2). This level of identity between samples has previously only been seen in the case of sub-clones picked or isolated from a common parental virus stock^30–32^.

**Table 1:** Sequencing statistics for R-13 and N-7 strains of HSV-1.

HSV-1 strain	Paired-end read length	Raw reads	Used for assembly	Genome length	Average depth (X-fold)	GenBank Accession
R-13	300 bp	3.1 × 10^6^	2.4 × 10^6^	151,636	7,626	KY922718
N-7	300 bp	4.3 × 10^6^	3.6 × 10^6^	151,765	11,640	KY922719

.

**Table 2:** Pair-wise DNA identity and variant proteins between HSV-1 consensus genomes.

Comparisons between strains	DNA identity	Intergenic		Genic		
		in/dels (# events)	SNVs	in/dels (# events)	Synonymous SNVs	Non-synonymous SNVs
N-7 vs. R-13	98.9%	16	2	2*	0	0

^*^54 bp insertion in VP1/2 (UL36) and a 6 bp insertion in ICP4 (RS1) in N-7, relative to R-13.

For the 1.1% of the genome that did differ between HSV-1 isolates R-13 and N-7, we categorized these differences as genic (coding) vs. intergenic, and grouped insertions or deletions (in/dels) vs. single-nucleotide variations (SNVs) (Table 2). We found no SNVs in any coding sequence, and only two in intergenic regions. For the in/dels, we calculated the minimum number of insertion or deletions events that could have led to the observed differences. For instance, a three base pair insertion was counted as one in/del event. There were a total of 18 in/del events, of which only two occurred in coding regions. These included an 54 base pair (18 amino acid (AA)) in/del in the gene encoding the ubiquitin-specific protease, VP1/2 (UL36), and a 6 base pair (2 AA) in/del in the transcriptional regulator protein, ICP4 (RS1) (Table 2). The insertion in VP1/2 is present in the C-terminal region of the protein, which contains an extended array of ‘PQ’ tandem repeats. Sequence length fluctuation in this region has been documented in HSV and other alphaherpesviruses such as varicella zoster virus (VZV), Marek’s disease virus (MDV), and pseudorabies virus (PRV)^33–37^. Similarly, the insertion observed in ICP4 is also present in a short sequence repeat in a functionally uncharacterized domain of the protein^38^. In both cases, the N-7 viral genome from the son contains the longer sequence (i.e. has an insertion) relative to the R-13 (father’s) genome.

### Comparison of father and son isolates to other HSV-1 strains

To place the HSV-1 isolates from father (R-13) and son (N-7) in the context of previously sequenced HSV-1 isolates, we compared full-length genomes of R-13 and N-7 to all available HSV-1 genomes in GenBank (46 total; see Methods for full list). We investigated the relatedness of the genomes by constructing a network graph using SplitsTree (Supplementary Fig. S3). We observed that the father-son isolates R-13 and N-7 form a separate branch compared to all previously sequenced genomes, but are positioned in the tree graph between other Asian, European and African isolates. The phylogenetic separation of isolates based on geography has been previously described^31^.

To put the protein-coding features of HSV-1 isolates R-13 and N-7 in the context of prior work, we compared all protein (AA) sequences of these isolates to those encoded by 46 fully-sequenced HSV-1 strains. In this comparison, we found a total of 34 viral proteins containing AA variations in R-13 and N-7 that had never been observed before (see Supplementary Table 1 for a complete list**)**. This large number of unique residues may reflect the position of these isolates in the phylogenetic network, where they lie on a branch separate from other known strains. We also found 54 proteins with AA variations that differ from the HSV-1 reference genome (strain 17syn + , Genbank ID JN555585), but that have been observed previously in one or more of the other sequenced strains.

### Intra-strain variation: Detection of minority variants within each HSV-1 genome

**Figure 4:**
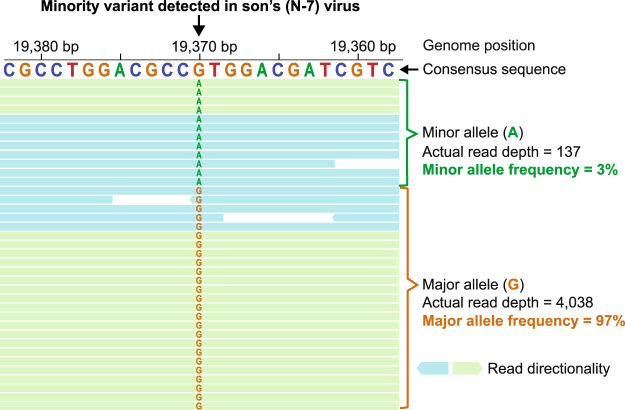
Intra-strain variation observed at a polymorphic locus in N-7 (son’s) viral genome, in the gene UL14. A low frequency non-synonymous variation was observed in the N-7 (son’s) viral genome at position 19,370, in the tegument protein UL14. This site has an A present in 3% of the viral sequence reads instead of the majority G allele (97%). The minority allele for UL14 encodes a valine to methionine change at residue 109; Met109 exists as the dominant allele in several independent isolates of HSV-1 (see text for details). While the UL14 coding sequence is encoded on the reverse strand of the reference genome for HSV-1, it is depicted here in forward orientation to enable codon reading from left to right. Actual read depth of each variant is indicated above. A subset of the alignment of Illumina sequencing reads to the N-7 consensus genome is shown here, with the position and consensus sequence shown in the top row. Sequence read orientation is depicted as aqua and green, with directional arrows. Areas with no letter shown have 100% agreement with the consensus nucleotide; the letters are left out for clarity.

Comparison of the consensus genomes of the HSV-1 isolates from father (R-13) and son (N-7) provided a global view of the overall identity of these viral isolates. However as observed for HCMV and VZV^17,21,39^, intra-strain variation can exist within a viral population, or within a local niche or lesion of a given host. To assess the extent of genetic diversity within each viral isolate, we investigated whether the consensus genome of either R-13 or N-7 contained any minority variants, or nucleotide positions where HTS data indicated more than one possible allele. This allowed us to detect specific nucleotide bases (loci) for which another allele was present; these minor differences would otherwise be missed in a comparison of the majority-genotype (consensus level genome) as seen in Table 2. We observed 59 minority variants in the R-13 genome and 48 in the N-7 genome (Supplementary Table 2). Most of the minor alleles observed in R-7 and N-13 genomes occurred at a low frequency or/and were intergenic. These minority variants were neutral and unlikely to have a major effect on viral fitness. These results are consistent with recent findings in HCMV, where it was shown that the vast majority of these minority variants observed were neutral^40^. Although rare, the minority variants that occurred in coding regions were non-synonymous, but with very low frequency alleles. The N-7 isolate harbored a 3.39% frequency of a non-synonymous variant in UL14 (Fig. 4), and another at 2.4% frequency in US6. The minority allele for UL14 encodes a valine to methionine change at residue 109 (V109M), which exists as the dominant allele in several independent isolates of HSV-1 (strains F (USA), CR38 (China), and R62 (South Korea))^31^. Most of the observed minority variants in each genome were present in intergenic regions, and adjoined tandem repeats or homopolymers (Supplementary Fig. 4). Length variations at tandem repeats and homopolymers are common between strains of HSV-1^31,41,42^, and have been documented in select coding regions as well (e.g. UL30 polymerase and TK)^43^.

## Discussion

Here we provide the first-ever insights on viral inter- and intra-strain variation during familial transmission of HSV-1. These viruses appear indistinguishable in their phenotypes in culture and in an animal model of pathogenesis. Their clinical course in the infected father and son were similar, with both experiencing relatively severe gingivostomatitis at the time of initial infection (as noted by each mother), followed by six to eight recurrences per year thereafter. The randomly selected HSV-1 isolates from each individual were nearly identical to each other at the genomic level, despite being separated by over four decades, a horizontal transmission event, and multiple rounds of latency and reactivation. This exceptional level of genomic identity contrasts with that observed for *in utero* transmission of HCMV, which was found to include both a bottleneck and a subsequent expansion of viral diversity^17,18,21,44^. However, HCMV and HSV-1 differ in their pathogenesis. HCMV is known to infect wider range of cell types, host organs, and undergo widespread viral dissemination *in vivo* as compared to HSV-1^45^. Furthermore, HCMV latency occurs in hematopoietic cells that undergo cell division, whereas the latent reservoir of HSV-1 in neurons does not undergo cell division. These distinctions may contribute to the observed differences in their level of intra-host variation.

The observation of nearly-perfect preservation of these viral genomes during familial transmission leaves open the question of where the observed diversity between clinical isolates of HSV-1 arises. One possibility (i) is that inter-strain diversity is the result of a slow mutation rate and variations that have accumulated over millions of years, with millions of clades of virus circulating around the planet. In this case the minority variants detected in the N-7 (son’s) viral genome may represent the fodder for evolutionary selection. Similarities in HSV-1 isolates from the same geographic regions suggests that selection for fitness adaptations to the local human populations may have occurred. However these geographic patterns could also be the result of founder effects and sequestration of human movement in early millennia.

Another possible explanation (ii) for the observed inter-strain diversity of HSV-1 isolates is that horizontal transmission between unrelated hosts may induce greater selection for mutations than familial transmission. Sexual partners are unlikely to have matched immune selection for a newly transmitted HSV-1 isolate, and MHC alleles have been found to influence symptoms and severity of HSV disease^46,47^. This hypothesis suggests that if a population of viruses are transmitted to a new unrelated host, then immune pressure over time in the new host could select for a viral population that differs from the one found in the original source.

Yet another possibility (iii) is that hosts with multiple HSV-1 infections or exposures may provide an opportunity for recombination to occur, with a concomitant rise in resulting variation of viruses shed by these individuals. Although rare, variations in the HSV-1 population(s) within an infected individual have been documented previously^32,48,49^. Analyses of HSV-1 genomes by RFLP or full genome comparison suggests that recombination between viral genomes has been rampant over historical time^31,50–54^. Finally, it is possible (iv) that different modes of transmission may affect the amount of variation transferred from a source individual, thus impacting the subsequent selection and fixation of these variants. For instance, it has been observed that shedding from lesion sites is associated with a high copy number of detectable viral genomes, whereas asymptomatic shedding has far fewer detectable genomes^55,56^. This suggests that asymptomatic shedding, which is a recognized source of transmission between individuals^57,58^, may represent a greater genetic bottleneck than transmission via contact with lesions.

In summary, using an animal model and HTS, we have shown that HSV-1 preserves its genetic and phenotypic characteristics during familial transmission. These approaches serve as a framework for future transmission studies using clinical isolates of HSV-1. Analysis of additional transmission events — particularly between unrelated individuals — will be necessary to assess how and where HSV-1 diversity arises.

## Methods

### Isolate acquisition and stock generation

Human Subjects were not recruited as part of this study. The samples described here were obtained through a virology laboratory at the Cincinnati Children’s Hospital Medical Center. Informed consent was obtained from individuals contributing the viral samples. Once cultured, the viruses were coded and non-identifiable, and were not human tissues. HSV-1 isolates were collected from the father and the son on separate occasions, two years apart. For each isolate acquisition, a sterile swab was used to obtain virus from a lip vesicle during a recurrence. The swab was placed in sterile media and transported on ice to the laboratory, where a portion of the virus containing media was directly absorbed onto a rabbit skin cell (RSC) monolayer. This first passage was aliquoted and used to generate a “Pass 2” stock that was utilized for all phenotypic studies. These new isolates were named according to recent recommendations, as outlined by Kuhn *et al*. (23). We use shortened forms of these names, R-13 (father) and N-7 (son) throughout the manuscript. The full names for these isolates are HSV-1/Cincinnati, USA/1995/R-13 and HSV-1/Cincinnati, USA/1993/N-7.

### Animal studies

All procedures in mice were performed as approved by the Children’s Hospital Institutional Animal Care and Use Committee (protocol# IACUC2013-0162) and were in compliance with the *Guide for the Care and Use of Laboratory Animals*. Animals were housed in American Association for Laboratory Animal Care-approved quarters. Male outbred Swiss Webster mice (22–25 grams in weight) obtained from Harlan Laboratories (now Envigo) were used for these studies. Prior to infection, mice were anaesthetized by intraperitoneal injection of sodium pentobarbital (50 mg/kg of body weight). A 10 μL drop containing 2 × 10^5^ PFU was placed onto each scarified corneal surface as detailed previously^29^.

### Virus replication *in vivo*

At the indicated times post infection (pi) infected mice were euthanized and tissues, including eyes, trigeminal ganglia (TG), and brains from three mice from each inoculation group were individually assayed for virus as previously detailed^24^. Isolation and quantification of total DNA from TG and quantification of total viral genomes by real time PCR using primers to the thymidine kinase region was performed as detailed previously^59^.

### Reactivation studies

Latent HSV was induced to reactivate in the ganglia of mice *in vivo* using hyperthermic stress (HS) (22). At 22 hours post induction, TG were assayed for infectious virus as detailed previously^24,25^. For *in vitro* explant reactivation studies, latently infected ganglia were aseptically removed and placed into MEM supplemented with 5% newborn calf serum. These were incubated at 37 °C in a 5% CO_2_ incubator. At the indicated times post explant, ganglia were homogenized and assayed for infectious virus as for reactivation *in vivo* ^60^.

### Antibodies and Immunohistochemistry

Whole TG were fixed in 0.5% formaldehyde for 2 hours, rinsed in PBS and post fixed in DENT’s fixative (80% methanol, 20% dimethylsulfoxide (DMSO)). Whole ganglia immunohistochemistry utilized a primary rabbit anti-HSV (AXL237, Accurate) at a 1:1000 dilution, and a secondary HRP-labeled goat anti-rabbit (Vector), at a 1:750 dilution. These methods have been detailed extensively in previous reports^26,27,59,61,62^.

### Virus culture and DNA isolation for HTS

Master stocks of the virus were prepared by infecting MRC-5 (ATCC®, CCL-171) human fetal lung fibroblast cells grown in Eagle’s Minimum Essential Media (EMEM:Sigma-Aldrich) at an MOI of 0.1. Viral stocks were harvested when significant cytopathic effect (CPE) was observed. Master stocks were tittered by limiting dilution assay on Vero cells (ATCC®, CCL-171) to calculate plaque-forming units (PFU) for each stock. For isolation of viral DNA, MRC-5 cell cultures were infected at an multiplicity of infection (MOI) of 10 and viral genomic DNA (gDNA) was isolated using previously described methods for the isolation of viral nucleocapsid DNA^63^.

### Southern blot

The genomic structures of the clinical isolates were analyzed by DNA (Southern) blot analysis^64,65^ and compared to four commonly-used HSV-1 strains (17syn + , McKrae, KOS(M), and F) and 10 unrelated clinical isolates. Genomic DNA was cleaved with BamHI, transferred to 0.8% agarose gels, blotted, and probed with a cosmid clone insert that was ^32^P-labeled using a Rediprime kit from Amersham. The cosmid clone insert matches the HSV-1 strain 17 genome from positions 24,698 to 64,405 (~40 kb). Blots were developed and analyzed on a Storm phosphorimager and quantified with GelQuantNet software.

### Illumina high-throughput sequencing

Sequencing libraries for each of the isolates were prepared using the Illumina TruSeq Nano DNA Sample Prep Kit, according to the manufacturer’s recommended protocol for sequencing of genomic DNA. The target DNA fragment size selected for library construction was 550 base pairs (bp). All the samples were sequenced on an in-house Illumina MiSeq using version 3 chemistry to obtain paired-end sequence fragments of 300 × 300 bp. Base calling and image analysis was performed with the MiSeq Control Software (MCS) version 2.3.0.

### *De novo* assembly of consensus genomes

HSV-1 genomes were assembled using a recently described viral genome assembly (VirGA) workflow^30^. Briefly, VirGA combines quality control preprocessing of reads, *de novo* assembly, genome linearization and annotation, and post-assembly quality assessments. HSV-1 strain 17 (GenBank Accession: JN555585) was used as comparator for the reference-guided portion of viral genome assembly in VirGA. GenBank accessions are listed in Table 1.

### Consensus genome comparison and phylogenetic analysis

Trimmed versions of the genomes lacking the terminal repeats were used for consensus genome comparison and for intra-strain polymorphism detection (below). We used the trimmed format of the genome to avoid over representation of the repeat regions during comparison. Clustalw2 (43) was used to construct pairwise global nucleotide alignments between whole genome sequences, and pairwise global amino acid alignments between open reading frames. These alignments were utilized by downstream custom Python scripts to calculate percent identity, protein differences, and genomic DNA variation between samples. Phylogenetic networks were constructed using SplitsTree4^66^ using the uncorrected_P distance, and all gaps were ignored. GenBank accession numbers and publications describing previously sequenced isolates are as follows: 17 (JN555585, NC_001806)^12,13^; F (GU734771)^36,67^; H129 (GU734772)^36,68^; KOS (JQ673480, JQ780693)^69,70^; McKrae (JQ730035,JX142173)^37,71,72^; HF10 (DQ889502)^73^; KOS63 (KT425110), KOS79 (KT425109)^32^; India (KJ847330); L2 (KT780616), SC16^74^; MacIntyre (KM222720)^75^; CJ970 (JN420341.1), CJ311 (JN420338.1), 134 (JN4000093.1)^76^; RE (KF498959); 160/1982 (LT594192), 132/1998 (LT594457), 1319/2005 (LT594108), 1394/2005 (LT594111), 66/2007 (LT594110), 20/2007 (LT594109), 369/2007 (LT594112), 2158/2007 (LT594106), 3083/2008 (LT594107), 172/2010 (LT594105)^77^; CR38 (HM585508), EO3 (HM585509), E06 (HM585496), E07 (HM585497), E08 (HM585498), E10 (HM585499), E11 (HM585500), E12 (HM585501), E13 (HM585502), E14 (HM585510), E15 (HM585503), E19 (HM585511), E22 (HM585504), E23 (HM585505), E25 (HM585506), E35 (HM585507), R11 (HM585514), R62 (HM585515), S23 (HM585512), S25 (HM585513)^31^.

### Intra-strain minority-variant detection

Minority variant loci or polymorphic positions within each consensus genome were detected using parameters that aimed to distinguish truly polymorphic sites from errors produced during sequencing or during *de novo* assembly of these genomes. VarScan v2.2.11^78^ was used to detect variants present within each consensus genome. To aid in differentiating true variants from potential sequencing errors^79^, the following variant calling parameters^39^ were used: minimum variant allele frequency ≥0.02; base call quality ≥20; read depth at the position ≥100; independent reads supporting minor allele ≥5. Polymorphisms with directional strand bias ≥90% were excluded. The variants obtained from VarScan were then mapped back to the genome to understand their distribution and mutational impact using SnpEff and SnpSift^80,81^. Polymorphic positions were visually assessed and hand-curated (Supplementary Table 1) to label those that bordered homopolymeric tracts or short sequence repeats (tandem repeats), since both can induce local mis-alignment of reads and lead to imprecise local polymorphism detection.

### Data Availability

Viral genomes sequenced during this study are included in GenBank under the following Accessions: KY922718, R-13 (full name: HSV-1/Cincinnati, USA/1995/R-13; KY922719, N-7 (full name: HSV-1/Cincinnati, USA/1993/N-7). All other data generated or analyzed during this study are included in this published article (and its Supplementary Information files).

## Electronic supplementary material

Supplementary Figures and Table S1

Supplementary Table S2
