# Supplementary material for: Inferred father-to-son transmission of herpes simplex virus results in near-perfect preservation of viral genome identity and in vivo phenotypes
Source: Sci Rep. 2017 Oct 20;7:13666. doi: 10.1038/s41598-017-13936-6 (PMC5654476; doi:10.1038/s41598-017-13936-6)
Supplement: Supplementary file 1 — Supplementary Figures and Table S1 [file 41598_2017_13936_MOESM1_ESM.pdf]

Inferred father-to-son transmission of herpes simplex virus results in near-perfect preservation of viral genome identity and *in vivo* phenotypes

Utsav Pandey[1], Daniel W. Renner[1], Richard Thompson[2], Moriah L. Szpara[1*], Nancy Sawtell[3]

[1]Department of Biochemistry and Molecular Biology, Center for Infectious Disease Dynamics, and the Huck Institutes of the Life Sciences, Pennsylvania State University, University Park, Pennsylvania 16802, USA

[2]Department of Molecular Genetics, Biochemistry and Microbiology, University of Cincinnati, Cincinnati, Ohio, 45229, USA

[3]Division of Infectious Diseases, Cincinnati Children's Hospital Medical Center, Cincinnati, Ohio, 45229, USA

***Corresponding Author**
Email: moriah@psu.edu

Supplementary Figures

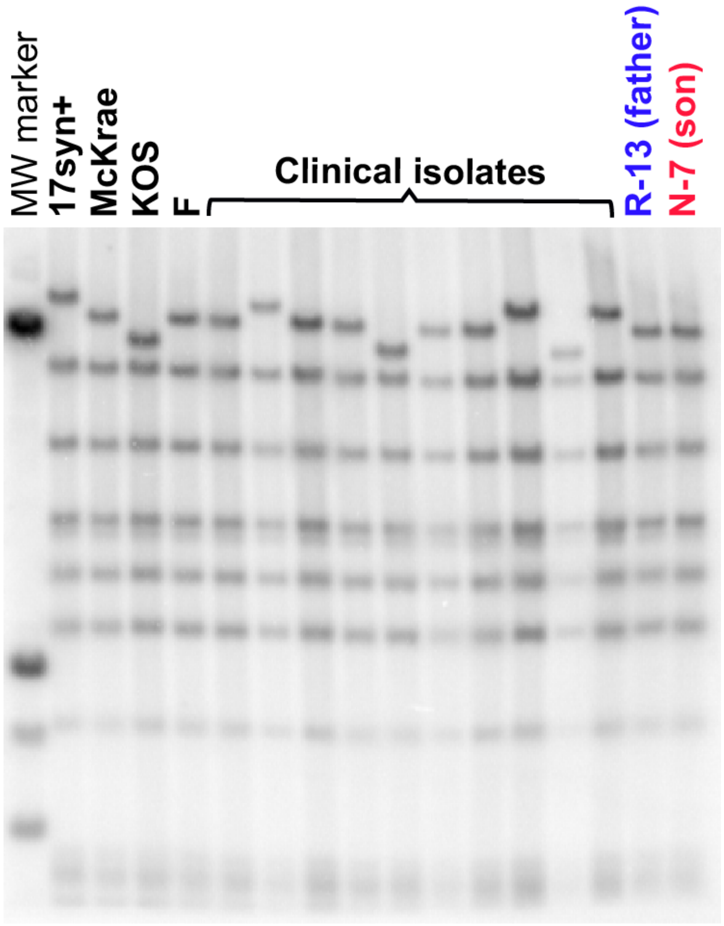

Supplementary Figure S1. Southern blot comparison of genetic variation in R-13 (father's) and N-7 (son's) isolates, relative to other strains of HSV-1.

The overall genomic structure of the father's and son's clinical isolates were analyzed by DNA (Southern) blot analysis and compared to four common laboratory strains 17syn+, McKrae, KOS(M), F, and ten different clinical isolates. Viral genomic DNA was cleaved with BamHI, gel-separated, and probed with a cosmid clone insert spanning 40 kbp of strain 17syn+ genome (see Methods for details). No major rearrangements or changes in fragment size were observed between the R-13 (father) and N-7 (son) isolates.

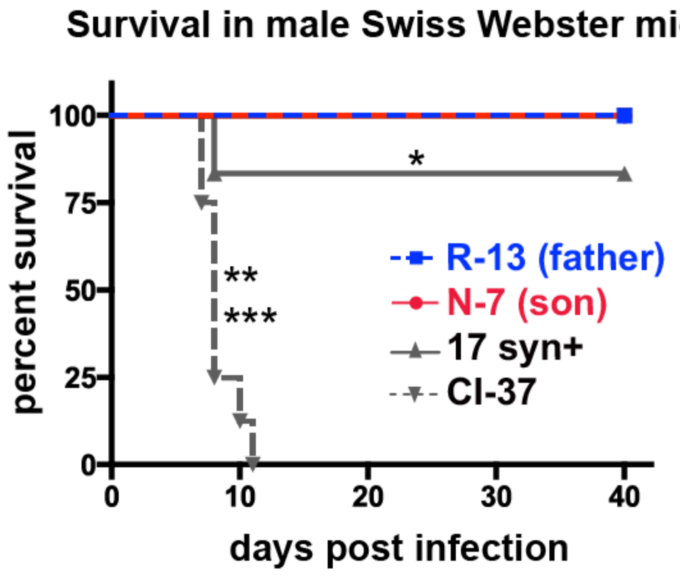

Supplementary Figure S2. No mortality was observed in Swiss Webster mice infected with either the father's (R-13) or the son's (N-7) isolate of HSV-1.

Swiss Webster Mice were infected via the ocular route with 2×10^5 PFU of R-13 (father's), N-7 (son's), 17syn+, and CI-37 HSV-1 (see **Methods** for details). Neither N-7 nor R-13 caused any death of mice through 40 days post infection, whereas 17syn+ and CI-37 caused 19% and 100% mortality respectively (R-13, 35/35 mice survived; N-7, 34/34 mice survived; CI-37 5/5 mice died; 17syn+ 13/16 mice died.). Mortality rate for isolate CI-37 was significantly different as compared to 17syn+ (ANOVA, ** $p<0.003$) and N-7 or R-13 (ANOVA, *** $p<0.0001$). The mortality rate for 17syn+ was also significantly different as compared to N-7 or R-13 (ANOVA, * $p<0.03$).

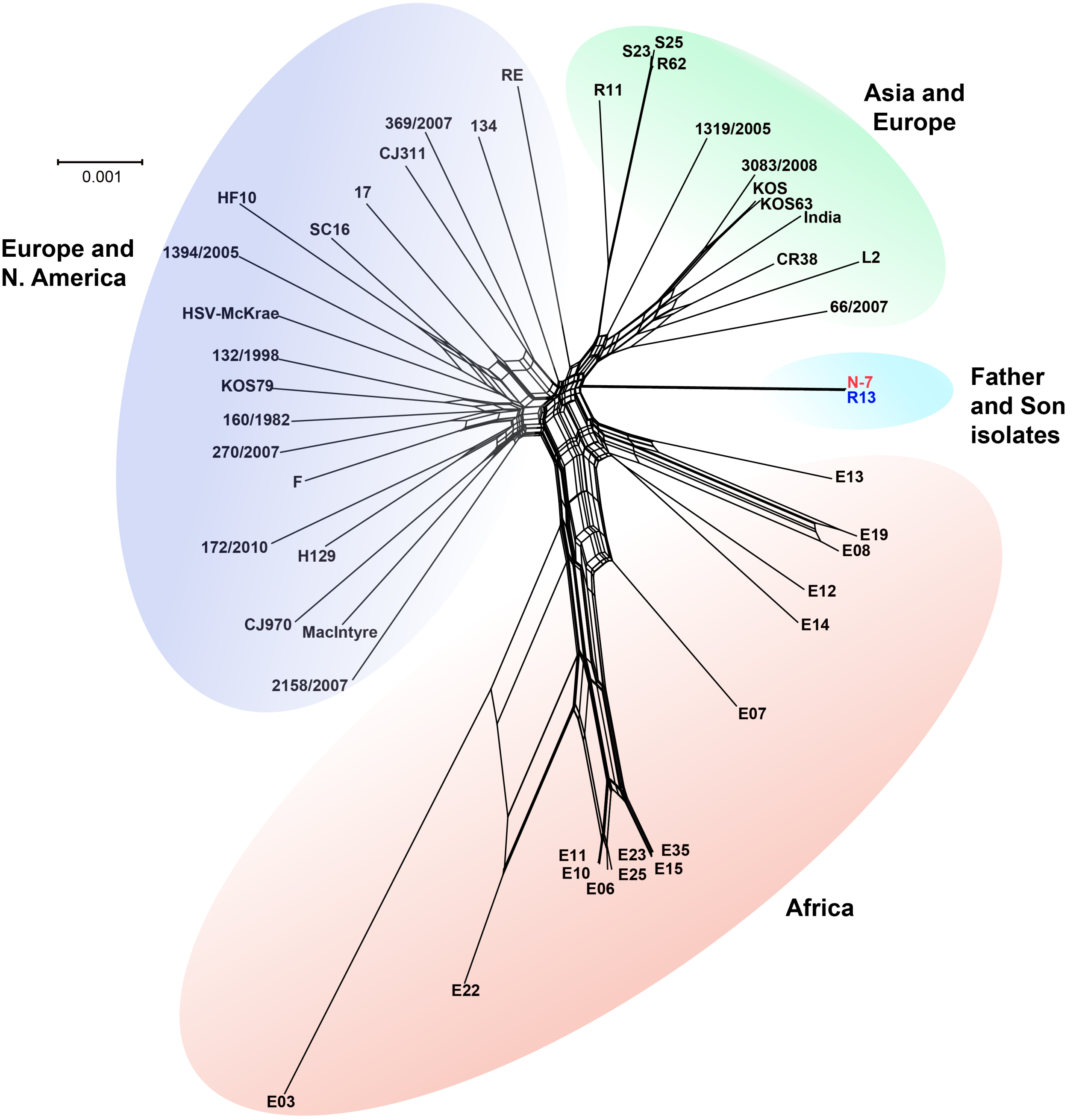

Supplementary Figure S3: A phylogenetic network showing genetic relatedness between isolates R-13 (father), N-7 (son) and previously sequenced HSV-1 isolates.

A phylogenetic network between isolates R-13 (father), N-7 (son) and all available complete HSV-1 genomes was constructed using SplitsTree4. The father and son isolates form a separate

branch compared to all previously sequenced HSV-1 genomes, with their branch localized between the Asian/European and African clusters. See Methods for a complete list of strain names and GenBank accessions for the HSV-1 genomes included in this analysis.

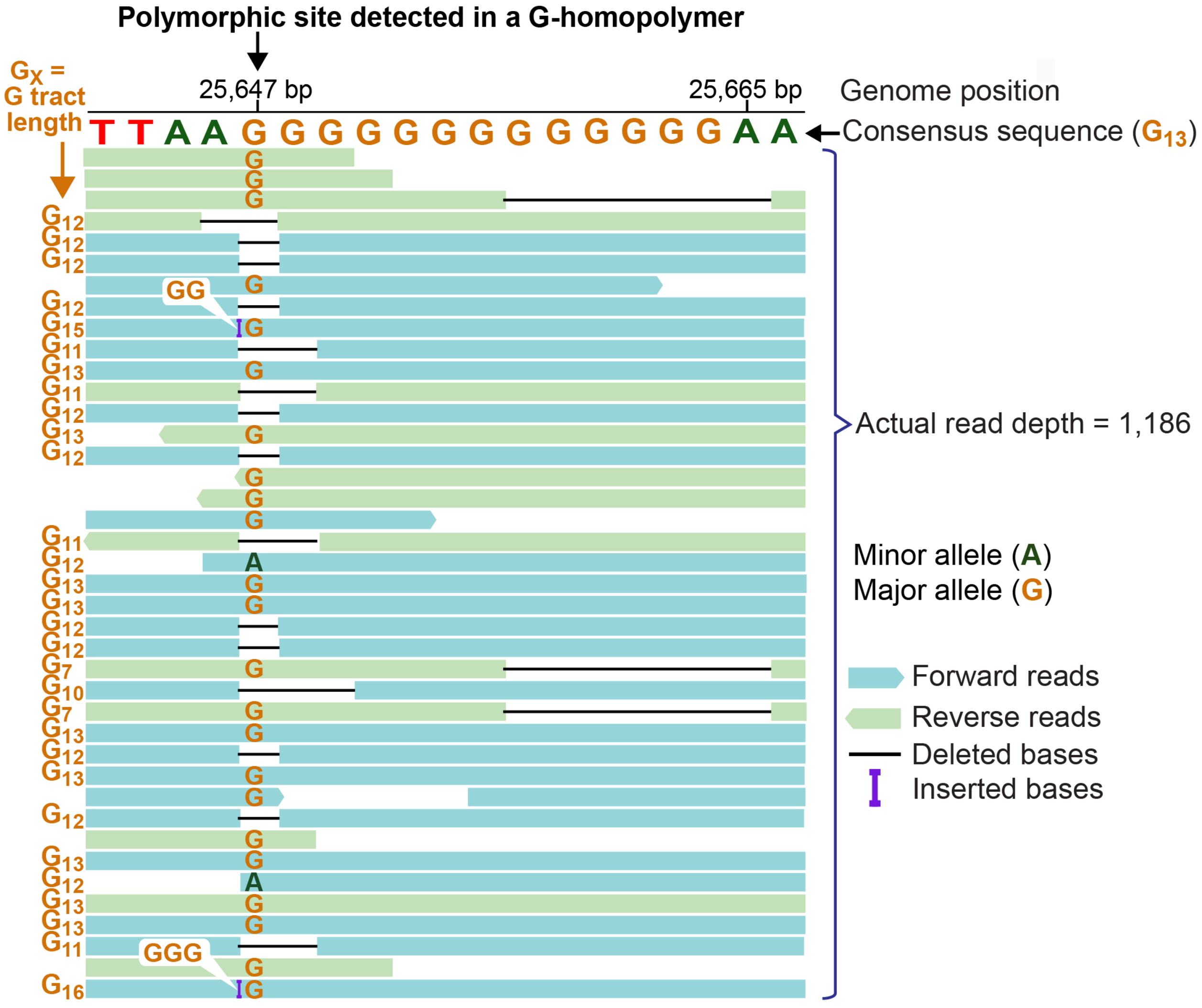

Supplementary Figure S4: Intra-strain variation observed at a polymorphic locus adjoining a homopolymeric tract, in an intergenic region of the HSV-1 isolate N-7.

A potential polymorphic locus was detected at position 25,647 of both the son's N-7 (7.8% minority allele), and the father's R-13 (4% minority allele) viral genomes. However inspection of the alignment of Illumina sequencing reads to the consensus genome (N-7 shown here) revealed that the polymorphic site detection resulted from a combination of small insertions or deletions in a homopolymeric tract of Gs in the consensus genome. A subset of the alignment of Illumina sequencing reads to the N-7 consensus genome is shown here, with the position and

consensus sequence shown in the top row. Actual read depth at the position is indicated above. Insertions relative to the consensus are shown as a blue "I" (labeled as GG or GGG), and deletions relative to the consensus are shown as a black horizontal line (range of 1-6 bp shorter) in the aligned sequence read. The length of the G-homopolymer tract is shown on the left, for those sequence reads that completely span the homopolymer tract. Homopolymer tract length cannot be inferred for reads that terminate within the G-tract; thus no length is listed for those reads. This position and other polymorphic loci that were detected adjacent to tandem repeats and homopolymeric tracts were flagged as such in Supplementary Table 2. Forward-oriented sequence reads are colored aqua, while reverse-oriented reads are colored green. Areas with no letter shown have 100% agreement with the consensus nucleotide; the letters are left out for clarity.

Supplementary Tables

Supplementary Table S1: Viral proteins with unique variations present only in isolates R-13 (father) and N-7 (son).

Protein	Mutation	Function
RL1 (ICP34.5)	Deletion of residues 92E,93P, P95Q	Blocks innate and adaptive immunity
RL2 (ICP0)	G76E	Viral gene expression, E3 ubiquitin ligase
RS1 (ICP4)	A1208S	Transactivator/repressor of viral transcription
UL2	E216K	Uracil DNA glycosylase
UL6	A395V	DNA entry into capsid
UL8	A183V	Encodes putative primase subunit of helicase primase
UL11	D37N	Membrane fusion during virus entry
UL12	D303Y, P30H, P10S	DNA exonuclease
UL16	P22S	Interacts with UL11 and UL21
UL22 (gH)	Q528K	Virion infectivity, cell-cell spread
UL24	A210V	Unknown
UL25	T339P	DNA encapsidation
UL26	G64V, A203V	Protease involved in capsid assembly
UL28	L184F, R720S	DNA encapsidation
UL29 (ICP8)	A319T	ssDNA binding protein necessary for DNA replication
UL30	R1229I	Catalytic subunit of DNA polymerase
UL34	W267C	Capsid exit from nucleus
UL36	H393Y, D987E	Ubiquitin specific protease
UL37	L1119F	Interacts with UL29 and UL36 to promote viral assembly
UL38	L349I	Minor capsid protein
UL39	L385M, E886A	Ribonucleotide reductase
UL42	R404G, P444S	DNA polymerase processivity factor
UL43	G22V, E/A253G	Unknown
UL45	A27T	Unknown
UL46	A8T, A266V	Regulates UL48 and UL47
UL47	A275G	Enhances activity of UL48 to induce α genes
UL52	D925G	Encodes primase subunit of helicase primase
UL54 (ICP27)	D263N, M339I	Inhibits host protein synthesis
UL55	A87V	Unknown
US1 (ICP22)	P108H	Regulatory protein
US3	A153T, V409A	Serine-Threonine kinase
US5 (gJ)	W7G, S41A	Blocks apoptosis of host cells
US8A	S27N	Virulence factor
US10	A211T	Unknown

Supplementary Table S2: Intra-strain variation -- polymorphic loci detected within the N-7 and R-13 consensus genomes.

This Excel table lists all potentially polymorphic loci detected in each viral genome. Loci that were detected by VarScan but did not pass the filtering criteria (e.g. those with only unidirectional read support) are included at the bottom of the file. For each polymorphic locus, the table lists the position in the genome, the major allele, the minor allele, the minor allele frequency, the number of reads supporting the major allele on the forward strand, the number of reads supporting the major allele on the reverse strand, the number of reads supporting the minor allele on the forward strand, the number of reads supporting the minor allele on the reverse strand, the percent of reads supporting minor allele on forward strand, the percent of reads supporting the minor allele on the reverse strand, the type of variation (e.g. intergenic, genic non-synonymous, or genic synonymous), and the gene affected (if any). The final column includes a hand-curated notation on whether the polymorphic locus was located adjacent to a homopolymer or tandem repeat, which could induce mis-alignment and imprecise polymorphism detection, or whether it is likely to be a minority variant (polymorphic locus).
